# Impact of clonally-related *Burkholderia contaminans* strains in two patients attending an Italian cystic fibrosis centre: a case report

**DOI:** 10.1186/s12890-019-0923-6

**Published:** 2019-08-29

**Authors:** Daniela Savi, Serena Quattrucci, Maria Trancassini, Claudia Dalmastri, Riccardo V. De Biase, Marta Maggisano, Paolo Palange, Annamaria Bevivino

**Affiliations:** 1 Department of Public Health and Infectious Diseases, Adult Cystic Fibrosis Centre, “Sapienza” University of Rome, V.le Universita’ 37, 00185 Rome, Italy; 2 Cystic Fibrosis Unit, Bambino Gesù Children’s Hospital, Rome, Italy; 3 Department of Pediatrics, Cystic Fibrosis Centre, “Sapienza” University of Rome, Rome, Italy; 4 Department of Public Health and Infectious Diseases, Laboratory of Microbiology, Policlinico Umberto I Hospital, Rome, Italy; 5 Department for Sustainability, Italian National Agency for New Technologies, Energy and Sustainable Economic Development, ENEA C.R Casaccia, 00123 Rome, Italy; 6 Sant’Andrea Hospital, Sapienza University of Rome, Rome, Italy

**Keywords:** Cystic fibrosis, *Burkholderia contaminans*, Multi locus sequence typing, Molecular typing, Lung function, Coinfection

## Abstract

**Background:**

*Burkholderia contaminans* is one of the 20 closely related bacterial of the *Burkholderia cepacia* complex, a group of bacteria that are ubiquitous in the environment and capable of infecting people with cystic fibrosis (CF). This species is an emerging pathogen and it has been widely isolated from CF patients in Argentina, Spain, Portugal, Australia, Canada, USA with a low prevalence in Ireland, France, Russia, Switzerland, Czech Republic, and Italy. This is the first report of *B. contaminans* affecting two Italian CF patients attending the same CF Centre. We correlate *B. contaminans* colonisation with lung function decline and co-infection with other clinically relevant CF pathogens.

**Case presentation:**

*B. contaminans* was identified by Multi Locus Sequence Typing in routine sputum analysis of two Caucasian CF women homozygous for Phe508del CFTR mutation. Sequence Type 102 was detected in both strains. It is known that *B. contaminans* ST102 was isolated both from CF and non-CF patients, with an intercontinental spread across the world. Random Amplified Polymorphic DNA analysis revealed the genetic relatedness between the two strains. We examined their susceptibility to antimicrobial agents, comparing the latter with that recorded for other *B. contaminans* isolated from different countries. We also described key virulence factors possibly linked with a clinical outcome. Specifically, we attempted to correlate colonization with the incidence of acute exacerbation of symptoms and lung function decline.

**Conclusions:**

This case presentation suggests that acquisition of *B. contaminans* ST102 is not directly associated with a lung function decline. We retain that the presence of other CF pathogens (i.e. MRSA and *Trichosporon*) along with *B. contaminans* ST102 might have contributed to the worsening of clinical conditions in our CF patients. The circumstances leading to the establishment of *B. contaminans* ST102 infections are still unknown. We highlight the importance to proper detect and typing bacteria implicated in CF infection by using molecular techniques.

## Background

Bacteria of the *Burkholderia cepacia* complex (BCC) are known opportunistic nosocomial pathogens causing severe respiratory infections in immunocompromised hosts and in Cystic Fibrosis (CF) patients [1]. BCC bacteria are associated with a poor prognosis, a rapid decline in lung function and reduced median survival [2]. Their heterogeneous intrinsic resistance to a wide range of antibiotics as well as their ability to develop further resistance during therapy make the treatment of BCC infections particularly difficult in CF [3]. The development of molecular tools has improved both the genetic characterization of cystic fibrosis transmembrane conductance regulator (CFTR) gene and the BCC taxonomy knowledge, properly identifying isolates at species and lineage level [4, 5]. Actually, the group is known to encompass at least 20 closely related species found in many niches of both natural and clinical environments, whose laboratory identification can often prove difficult [6–11].

Epidemiological studies examining relationships between respiratory infection and clinical outcomes have defined the contributions of BCC species to CF lung disease. Over the 20 formally named species within the complex, *Burkholderia multivorans* (genomovar II) and *Burkholderia cenocepacia* (genomovar III) together account for approximately 85–97% of all BCC infections in CF [12]. Although it has been well demonstrated that lung function declines more rapidly in CF patients infected with *B. cenocepacia* [12], also *B. multivorans* and *Burkholderia dolosa* have been associated with severe outcomes in CF [13–16]. Over the past several years, potential novel BCC species have been recovered with greater frequency from CF respiratory secretions but predicting prognosis after their infection in CF is challenging. Among the BCC species, a growing clinical interest concerns *Burkholderia contaminans.* Isolates of both clinical and environmental origin previously identified as taxon K and included in Multilocus Sequence Typimg (MLST) groups I and II, were identified as *B. contaminans* [9], referring to the metagenome that was recovered from the Sargasso Sea (the so-called *Burkholderia* SAR-1 metagenome) [17]. In addition to forest soil and river water [9], *B. contaminans* has been recovered from contaminated pharmaceutical products [9] and healthcare facilities where it has caused outbreaks [18, 19].

Over the past decade, *B. contaminans* is increasingly associated with CF [20] and with hospitalized non-CF patients [21, 22]. *B. contaminans* is frequently isolated from CF respiratory samples in Ibero-America countries [23, 24] indicating a large and characteristic geographic distribution of considerable concern. As reported in Multilocus Sequence Typing (MLST) database website, *B. contaminans* was also detected in Italy [25] and named as LMG 23253 (id: 126) and R-19218 (id: 2695) [9]. To date, there are no epidemiological studies that revealed the presence of other *B. contaminans* strains in Italy. We describe for the first time a single Centre experience of two CF patients in which the *B. contaminans* was recovered from the sputum sample and typed by means of MLST and Random Amplified Polymorphic DNA. To elucidate the potential clinical impact of *B. contaminans*, we aimed to describe its key virulence factors possibly linked with a clinical outcome. Specifically, we attempted to correlate colonization with the incidence of acute pulmonary exacerbation and lung function decline.

## Case presentation

In May 2008 and in June 2008 two female CF patients were identified as *B. contaminans* positive at the Regional CF Centre of Policlinico Umberto I Hospital, Sapienza University of Rome, Italy, following MLST analysis as described by *Baldwin* et al. [5]. We collected demographic and clinical characteristics, chronic infections and CF comorbidity (pancreas insufficiency and CF-related diabetes) of both patients since they referred to our center. We also collected information on number of pulmonary exacerbations and exacerbation treatment in the preceding *B. contaminans* detection period and throughout the follow-up. Exacerbation events were ascertained through a review of electronic records and hospital charts.

**Fig. 1:**
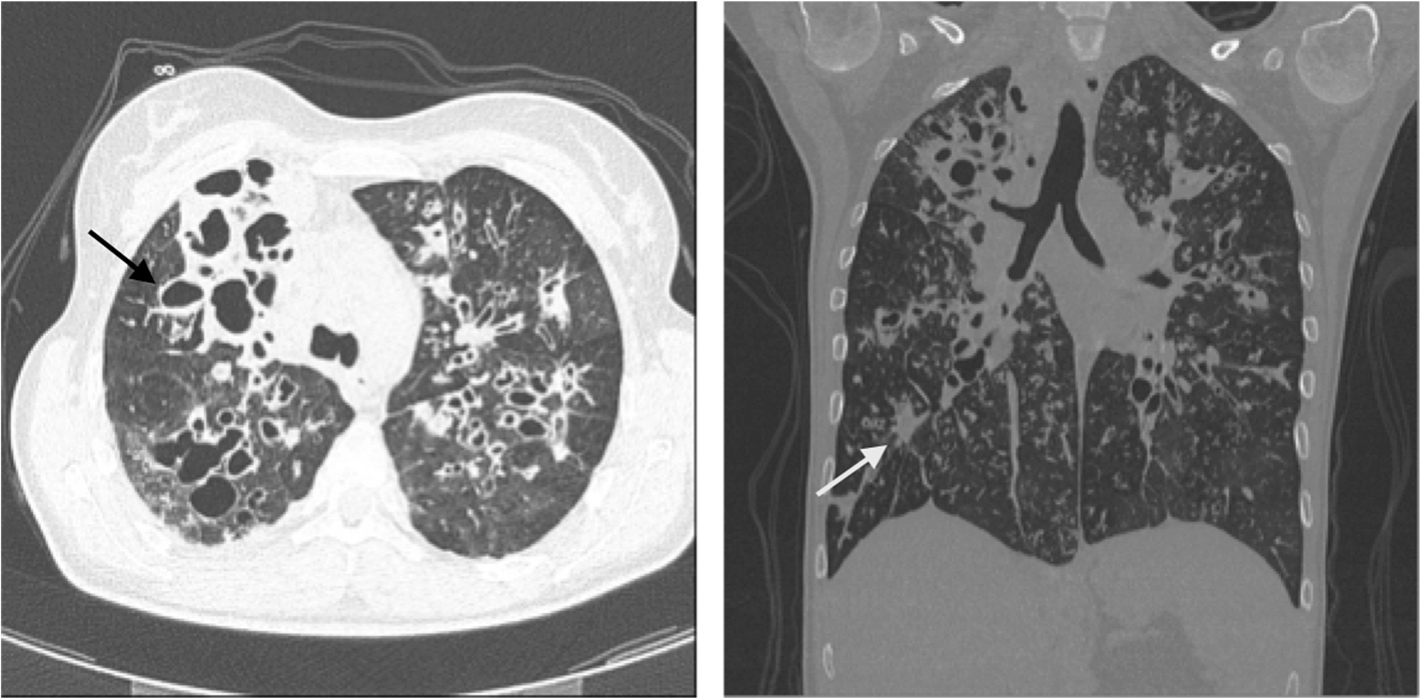
High Resolution Computed Tomography (HRCT) of the Patient 1′ Lungs. A) Axial and B) Coronal HRCT images showing severe bilateral bronchiectasis, mainly in the upper lobes (black arrow), with associated bronchial wall thickening and mucus retention in bronchial lumen. Bilateral ground-glass opacity with areas of consolidation (white arrow)

Patient 1 was a Caucasian 32-year-old CF woman diagnosed with CF shortly after birth, due to meconium ileus, CFTR genotype Phe508del/Phe508del. The patient demonstrated good compliance with her treatment regimen, complicated by insulin dependent diabetes mellitus CF related. From the age of 2 years she was infected with *Haemophilus influenzae* 10^4^ colony forming units (CFU)/ml, *Sthaphylococcus aureus* 10^4^ CFU/ml, *Stenotromonas malthophilia* 10^4^ CFU/ml, and *Aspergillus flavus* 10^4^ CFU/ml*.* In 1992 aged 16 years, she acquired *Methicillin-resistant Staphylococcus aureus* (MRSA) 10^4^ CFU/ml and became chronically infected. Her spirometry showed Forced Expiratory Volume in 1 s (FEV_1_) 2,47 L (72% of predicted) and Forced Vital Capacity (FVC) 3.01 L (78% of predicted). In August 2001, MRSA and a novel pathogen as *Trichosporon* 10^4^ CFU/ml have been recovered in her respiratory secretions. At this time, her FEV_1_ dropped from 2,18 L (64% of predicted) to 1,48 L (48% of predicted) and her body mass index (BMI) was 19,2 kg/m^2^. In May 2008, patient attended the CF Centre for the annual review. Microbiological analyses of sputum samples identified for the first time the presence of *B. contaminans* 10^5^ CFU/ml and showed also MRSA 10^6^ CFU/ml, *Achromobacter xylosoxidans* 10^4^ CFU/ml, *Trichosporon* spp. 10^5^ CFU/ml. Patient’s FEV_1_ was 43%, her full blood count at the time showed a mildly elevated white cell count of 10.98 10^9^/L, with neutrophil count of 4.69 10^9^/L and C-reactive protein (CRP) of 10.9 mg/dL. In August 2008, the patient was admitted for pulmonary exacerbation and was treated with a course on intravenous ceftazidime 50 mg per kg per dose every 8 h and oral sulfamethoxazole-trimethoprim combined with physiotherapy. At the discharge her lung function did not improve with FEV_1_ 1,21 L (40% of predicted) and FVC 1,86 L (52% of predicted). After 3 months she was admitted to hospital with poor nutritional status (BMI 18 kg/m2) and her FEV1 was 0,99 L (33% predicted), her lowest recorded. High resolution computed tomography (HRCT) showed severe bilateral bronchiectasis, mainly in the upper lobes with associated bronchial wall thickening and mucus retention in the bronchial lumen. Bilateral ground-glass opacity with areas of consolidation were also described (Fig. 1). It was decided to adopt a 2 weeks quadruple treatment strategy comprising IV ceftazidime 50 mg per kg per dose every 8 h, IV meropenem 40 mg per kg per dose, IV sulfamethoxazole-trimethoprim and chloramphenicol (500 mg every 6 h). Between January and July 2010, the patient required several hospital admissions for pulmonary exacerbations all of which were treated with a combination of IV meropenem 40 mg per kg per dose, IV tobramycin 300 mg once a day or IV Amikacin 500 mg bd, IV Linezolid 600 mg bd and IV Temocillin 2 g every 8 h. Oxygen supplementation was also started. In August 2010, the patient was admitted to hospital with acute dyspnea requiring increased oxygen supplementation and atrial fibrillation. A newsputum culture confirmed the presence of MRSA 10^4^ CFU/ml, *B. contaminans* 10^6^ CFU/ml and *Trichosporon spp* 10^6^ CFU/ml. She started antimicrobial treatment with IV meropenem, IV tobramycin, IV ceftazidime, IV voriconazole 200 mg bd, IV methylprenisolone 20 mg bd and IV verapamil. Her condition continued to worsen over the following 3 days with an elevated CRP 50 g/dL. She died 48 h later due to lung failure.

Patient 2 was a 34–year-old woman of Caucasian origin diagnosed with CF at the 1 year of age, due to respiratory symptoms. Her genotype is homozygous for the F 508del mutation. Between diagnosis and June 2008, the patient attended the Regional Cystic Fibrosis Centre in Rome, Italy. Her cough swabs and sputum cultures had yielded *Streptococcus viridans* 10^4^ CFU/ml and *S. aureus* 10^6^ CFU/ml. In 1984 she had also acquired, and had been treated for *Pseudomonas aeruginosa* 10^5^ CFU/ml, which recurred in 1989,1995 and 1998. During the observation period, FEV_1_ and FVC were regularly recorded and remained stable over time (FEV_1_ 3,12 L, 106% of predicted, FVC 3.4 L, 101% of predicted). Attempts to eradicate *P. aeruginosa* failed and she was deemed to be chronically infected in 1998. As the strain was sensitive to tobramycin following antibiotics susceptibility test, she was commenced on long-term anti-pseudomonal therapy comprising alternate months on-off of nebulised tobramycin twice daily. Since 1998 she was also chronically infected by MRSA 10^6^ CFU/ml. Although she had good compliance with her medications and adherence to physiotherapy, her lung function started to decline. In 2004 patient experienced admission for treatment of pulmonary exacerbation and her lung function on discharge were FEV_1_ 1,91 L (72% of predicted), FVC 2.43 L (79% of predicted), her lowest recorded. In June 2008, the patient presented for annual review and her FEV_1_was 1,7 L (65% of predicted), similar to previous annual review. MRSA was identified from her routine sputum sample. In addition, however, *B. contaminans* 10^5^ CFU/ml was also detected, representing the second incidence in our Centre. Her sputum was positive to *B. contaminans* 10^4^ CFU/ml again in December 2008 and to date it has never been detected. Between *B. contaminans* isolation and February 2017, the patient had experience two pulmonary exacerbation treated with oral antibiotics and both her nutritional status and pulmonary function remained stable. She commenced Lumacaftor/Ivacaftor therapy in February 2017. During the first year of treatment, the patient did not have exacerbations. Her last spirometry values increased with FEV_1_ 2.12 L (87% of predicted) and FVC 2.69 L (92% of predicted).

**Fig. 2:**
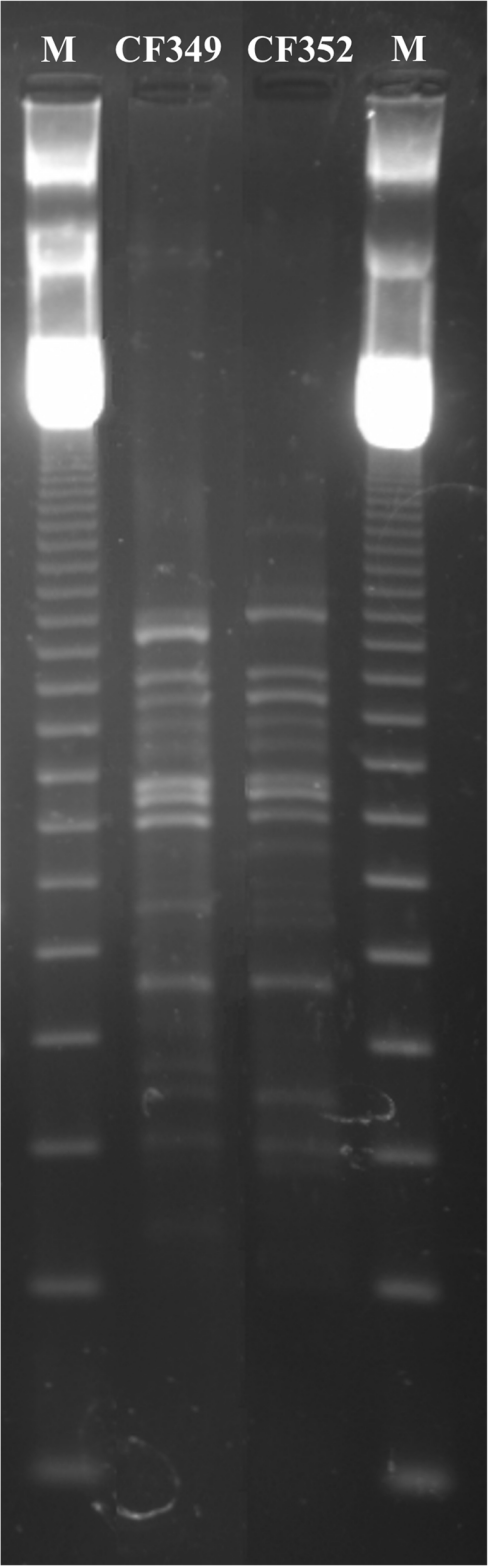
Random amplified polymorphic DNA (RAPD) fingerprints of *B. contaminans* isolates generated using primer 270. *Lane M,* 123-bp molecular size marker ladder. On the left, *B. contaminans,* CF 349 strain, recovered from Patient 1 in May 2008. On the right, *B. contaminans,* CF 352 strain, recovered from Patient 2 in June 2008

In both cases, *B. contaminans* was isolated from sputum samples collected by the patients through spontaneous expectoration during routine visits at our CF Centre. Samples were routinely processed according to standard CF microbiological procedures [26]. Samples were liquefied by the addition of Sputasol (Oxoid Ltd. Hampshire) at a 1:1 dilution (v/v) and incubation at 37 °C for 1 h to reduce the viscosity of the sputum. Ten-fold dilutions of treated samples (20 μl) were plated and cultured on common appropriate media for CF pathogens including *Burkholderia cepacia* selective agar medium (BCSA) (Bio-Mérieux Marcy-l’Etoile, France). Plates were incubated at 35 °C for 24–48 h and then maintained at room temperature up to 5 days. The presumptive *B. cepacia* complex colonies were identified as BCC by API 20NE System (Bio-Mérieux Marcy-l’Etoile, France) and stored at − 80 °C. The identification of the bacterial isolate recovered from BCSA plates to the species status of *B. contaminans* was subsequently assessed following the BCC MLST scheme developed by Baldwin and colleagues [5]. Genomic DNA was extracted from a liquid pure culture using the cetyltrimethylammonium bromide (CTAB) method [27]. PCR reactions and conditions were performed as described by Baldwin and colleagues [5] for seven independent housekeeping loci (*atpD*, *gltB*, *gyrB*, *recA*, *lepA*, *phaC*, *trpB*) were amplified by PCR as described by Baldwin and colleagues [5]. The amplification products were purified using Sephadex G-100 resin according to Cesarini and collegues [28]. The nucleotide sequences of the seven amplified genetic loci were determined using nested primers as described previously [5]. Sequencing reactions were prepared by using Applied Biosystem Big Dye® Terminator sequencing kit version 3.1, according to the manufacturer’s instructions and analyzed with an ABI PRISM 310 Genetic Analyzer Perkin-Elmer, at the ENEA Genome Research Facility DNA Sequencing Laboratory (Genelab, ENEA C.R. Casaccia, Italy). The forward and reverse sequences of each locus were aligned, trimmed to the desired allele length using SeqMan II (DNAStar software) [28], and compared with existing alleles in the BCC MLST database at http://pubmlst.org/bcc/ [29], in order to assign the isolate allelic profile obtained with a sequence type (ST), indicative of each genetically distinct strain [5]. The taxonomic status of isolates was determined by ST searching against the MLST database [28]. The bacterial isolate of the Patient 1, named CF 349, resulted to have the Sequence Typing (ST) 102, showing the following allelic profile: *atpD*: 64; *gltB*: 80; *gyrB*: 76; *recA*: 89; *lepA*: 105; *phaC*: 97; *trpB*: 70. The bacterial isolate of the Patient 2, named CF 352, resulted to have the Sequence Typing (ST) 102 as that found for CF 349 strain, showing the following allelic profile: *atpD: 64; gltB: 80; gyrB: 76; recA: 89; lepA: 105; phaC: 97; trpB: 70*. The two bacterial isolates, CF 349 and CF 352, were genetically typed by random amplified polymorphic DNA (RAPD) analysis as described by Mahenthiralingam et al. [30]. RAPD fingerprints were analyzed using the Quantity One software package (Bio-Rad Laboratories, Milan, Italy) as previously described [28]. Reproducibility was verified by RAPD fingerprinting each isolate at least four times in independent experiments. CF349 and CF352 isolates were genetically related since they exhibited the same genetic fingerprints differing by no more than two bands (Fig. 2). Antibiotic susceptibility test of CF349 and CF352 isolates is presented in Table 1. It was studied by employing the Kirby-Bauer disc-diffusion method on Mueller Hinton II Agar (Bio-Merieux Marcy l’Etoile, France) as recommended by the Clinical and Laboratory Standards Institute (CLSI) [31]. The results were determined by measuring the diameter of complete inhibition of bacterial growth. Zone diameters of susceptibility testing results were categorized as sensitive (S), intermediate (I), or resistant (R) based on the CLSI breakpoint criteria for *B. cepacia* for ceftazidime (CAZ), meropenem (MEM), imipenem (IMP), trimethoprim/sulfamethoxazole (SXT), and breakpoint criteria for *P. aeruginosa* for piperacillin/tazobactam (TZP), amikacin (AK), aztreonam (ATM), Ciprofloxacin (CIP), gentamicin (GEN) and tobramycin (TOB).

**Table 1:** Antibiotic resistance profile of *B. contaminans* CF349 and CF352 isolates

Isolate	Antibiotic resistance profile^a^									
	TZP	CAZ	MEM	IMP	ATM	GEN	TOB	AK	SXT	CIP
CF349^b^										
May 2008	S	S	R	R	I	R	R	S	R	I
December 2009	S	I	NA	R	R	R	R	R	R	R
March 2010	S	S	NA	R	R	R	R	R	R	R
July 2010	R	S	S	NA	R	R	R	R	R	R
CF352^c^										
July 2008	S	S	R	R	I	R	R	R	R	S
December 208	I	S	R	R	R	R	R	R	R	I

^a^*S* sensitivity, *I* intermediate resistance, *R* resistance. Antibiotic abbreviations: *TZP* piperacillin/tazobactam, *CAZ* ceftazidime, *MEM* meropenem, *IMP* imipenem, *ATM* aztreonam, *GEN* gentamicin, *TOB* tobramycin, *AK* amikacin, *SXT* trimethoprim/sulfamethoxazole, *CIP* ciprofloxacin

^b^CF349 = *B. contaminans* strain isolated from Patient 1 in May 2008

^c^CF352 = *B. contaminans* strain isolated from Patient 2 in June 2008

## Discussion

In this case report we described for the first time the genetic relationships between 2 *B. contaminans* isolates from two Italian CF patients attending the same CF Centre in the same period of time. We examined the clinical impact of this microorganism in two CF patients and the co-infection with other CF pathogens colonizing the patients’ respiratory tract. Strict infection control measures followed at the Regional CF Centre of Rome, Italy, including patient segregation, have reduced the epidemic spread but not eliminated new BCC infections, and CF patients may occasionally become infected by strains showing novel fingerprint types [32]. The present study reports the first case of *B. contaminans* acquisition in our CF Centre. MLST analysis performed on BCC strains collected from our patients did not reveal any other colonisation of *B. contaminans* during the outbreak period, in the previous years and up to date. This finding highlighted the continuing role of BCC species as opportunistic pathogens [33]. An accurate and rapid species identification within BCC is fundamental to manage BCC infections in CF patients in relation to appropriate therapy, infection control and lung transplantation. The development of molecular techniques, that has been improving knowledge in BCC taxonomy, enables to properly identify isolates that were previously difficult to be characterized, helping to individuate the strains responsible for novel acquisition [5]. The *Burkholderia cepacia* complex Multi Locus Sequence Typing website (https://pubmlst.org/bcc/) reveals the presence of 94 *B. contaminans* records and/or id, having 35 different STs*.* ST102 includes 20 strains isolated from environmental sources such as Sargasso sea [17], from sheep mastitis (milk) [34], and from human sources, including sputum and blood samples of CF and non-CF patients in North and South America, and Europe [25]. Some strains (AU7143 LMG23252) belonging to ST102 have been involved in widespread outbreaks in the United States and Brazil [25, 35]. Several cases of epidemic transmission of taxon K strains among CF patients have been reported in Italy [36], and in Portugal [37], and it is likely that environment may act as reservoir for novel BCC infections [38]. A positive selection of hypermutators, linked to antimicrobial resistance development, might be playing a key role in increasing *B. contaminans* adaptability to the CF-airway environment, facilitating the persistence of chronic lung infections [39]. As results of the segregation policies followed at the Regional Cystic Fibrosis Centre of Rome, CF patients harbour now unique BCC strains and the present study reports the first case of *B. contaminans* acquisition of closely-related strains by two patients. In the last few years, the potential clinical impact of infection with *B. contaminans* has been investigated [40]. There is evidence in CF that *B. contaminans* infections are often of transient nature [41] and thus can be perceived as respiratory tract colonization rather than true infection [40]. Only some patients develop chronic infection with long-term culture positivity [24]. However, some of the cases can get worse and result in fatalities.

The clinical experience about *B. contaminans* ST102 identified in our Centre was similar in both CF patients described in this report. Specifically, lung function decline and clinical deterioration were associated with the isolation of other CF pathogens along with *B. contaminans*. In effect, Patient 1 presented a deep decline of FEV_1_ and nutritional status in August 2001, when MRSA and a novel pathogen as *Trichosporon* spp. have been recovered in her respiratory secretions. At the time of first isolation of *B. contaminans*, lung function values did not change in comparison to those found 1 year before BCC colonization (FEV_1_ 43% predicted vs 42% predicted and FVC 54% predicted vs 56%). While the contribution of MRSA to CF lung disease has been well defined, the potential pathogenicity of *Trichosporon* spp. was recently defined [42]. In agreement with Patient 1, Patient 2 showed a FEV_1_ decline in association with MRSA respiratory infection. Transient colonization of the respiratory tract by *B. contaminans* was not associated with changes in clinical outcome measures. Both CF patients showed a link between infection and disease when other pathogens were recovered from culture rather than *B. contaminans* isolation. At the time of *B. contaminans* detection, both Patient 1 and Patient 2 were attending the same adult CF clinic. After the identification of *B. contaminans*, we implemented the strategy focused on reducing cross-infection risk (either via direct exposures or surface contamination), and involved clinic sterilization, reinforcement of strict isolation practices and education of both members of the CF team and patients. Containment appeared to have been effective as *B. contaminans* has not been isolated in our CF Centre since.

In conclusion, our results suggest that acquisition of *B. contaminans* strain was not associated with a decline in lung function and highlight the feature that circumstances leading to the establishment of *B. contaminans* infections are still unknown. We retain that several factors such as patients’ clinical characteristics and other pathogens associated with CF pulmonary exacerbations, along with *B. contaminans* acquisition, might have contributed to the worsening of general conditions in our CF patients. This study demonstrates that *B. contaminans* infection can occur, probably due to the wide diffusion of this species in the environment. Our data also support an environmental/hospital origin of *B. contaminans* infections. The detection and accurate identification of the novel BCC species in CF specimens are fundamental for the analysis of the clinical response of CF individuals to BCC infection and are expected to have a significant impact on future clinical management in relation to appropriate therapy, infection control and lung transplantation.

## Data Availability

Data and material are available on reasonable request.

## Abbreviations

BCC: *Burkholderia cepacia* complex
CF: Cystic fibrosis
FEV1: forced expiratory volume in 1 second
FVC: forced vital capacity
HRCT: High resolution computed tomography
MLST: Multilocus Sequence Typing
MRSA: Methicillin-resistant *Staphylococcus aureus*
RAPD: Random Amplified Polymorphic DNA
